# Immuno-Kachiks formula immunomodulates and ameliorates hepatic damage induced by monosodium glutamate in rats

**DOI:** 10.1016/j.heliyon.2024.e27076

**Published:** 2024-02-29

**Authors:** Geoffrey Kachiko, Anke Weisheit, Clement Olusoji Ajayi, Casim Umba Tolo, Jonans Tusiimire

**Affiliations:** a Pharm-BioTechnology and Traditional Medicine Center of Excellence (PHARMBIOTRAC), Mbarara University of Science and Technology, P.O. Box 1410, Mbarara, Uganda; b Faculty of Medicine, Mbarara University of Science and Technology, P.O. Box 1410, Mbarara, Uganda; c Department of Pharmacy, Mbarara University of Science and Technology, P.O. Box 1410, Mbarara, Uganda

**Keywords:** Immuno-Kachiks formula, Immune booster, Liver disease, Suppressed immunity, Monosodium glutamate

## Abstract

The immune system plays a vital role in controlling liver fibrosis and enhancing the pathogenesis of liver inflammation. Monosodium glutamate is a common flavor-enhancement food additive. This study evaluated the immunomodulatory and hepato-curative effects of the Immuno-Kachiks polyherbal formulation against monosodium glutamate-induced immune suppression and hepatic damage in rats. Monosodium glutamate was given orally at a 2000 mg/kg dose to male Wistar rats for three months to induce liver damage and immune suppression. After three months of successful induction, three groups were separately administered orally with Immuno-Kachiks formula at 400, 800, and 1500 mg/kg/day for 28 days. At the end of the treatment period, liver and blood samples were collected for histological and biochemical analysis. The lymphocyte count remained significantly low while the neutrophil count and the neutrophil-to-lymphocyte ratio increased significantly, despite the cessation of monosodium glutamate ingestion for 28 days. The Immuno-Kachiks formula (IKF) significantly increased the lymphocyte count, reduced the neutrophil-to-lymphocyte ratio, and normalized the neutrophil count. Neither monosodium glutamate nor the IKF significantly caused alpha-fetoprotein levels to rise or fall below normal. High doses (800 and 1500 mg/kg) of the Immuno-Kachiks formula significantly raised serum levels of aspartate aminotransferase, alkaline phosphatase, and total bilirubin. 1500 mg/kg of the IKF caused mild liver inflammation. The IKF restored the liver morphologic alterations observed in monosodium glutamate-induced liver damage in rats. The results suggest that the Immuno-Kachiks herbal formulation is a potential curative agent for early-stage liver damage and could restore suppressed adaptive immunity.

## 1 Introduction

Monosodium glutamate (MSG) is commonly added to soups, roasted meats, vegetables, and other restaurant foods to enhance flavor. Humans consume approximately 0.4–20 g of glutamate daily, mostly from protein-containing foods such as meat, cheese, legumes, and nuts [1,2]. Reports have revealed that human consumption of monosodium glutamate is on the rise and is expected to further increase by 6.2% by the year 2026 [3]. In addition to ingested glutamate, approximately 40–80 g of glutamate are synthesized per day, and the excess is stored in major organs such as the brain, muscles, and liver [4]. Excess glutamate is a known excitotoxin. Excitotoxins damage nerve cells by overstimulating neuron receptors. Excitotoxins cause inflammation, injury, or death of neurons [5].

Several reports have debated the safety of MSG consumption. The U.S. Food and Drug Administration, the European Food Safety Association, and the Joint FAO/WHO Expert Committee on Food Additives declared monosodium glutamate safe for consumption [6]. A study review by Zanfirescu et al. queried the methods used by studies reporting that MSG was harmful [6]. Their review highlighted that the doses used in those animal studies were far too high compared to the reported amounts of MSG consumed by humans. Later, in 2022, Hamad demonstrated that a dose of MSG as low as 5 mg/kg causes liver and spleen damage in test rats [7].

Another review by Banerjee et al. 2021 concluded that combining MSG with a high-lipid diet results in numerous diseases, including malignancies [2]. Additionally, additional human studies have reported that monosodium glutamate affects sex organs and often causes “*Chinese restaurant syndrome*,” which is characterized by metabolic disorders, neurotoxic symptoms, and obesity [8]. Despite the above differing perspectives, several studies have demonstrated that concurrent consumption of MSG with herbs has both protective and curative potential against different diseases induced by MSG in animals [9].

Herbs are food because they contain nutrients. Herbs are also drugs because they contain phytochemicals that are active against numerous disease conditions, including inflammation, liver damage, and suppressed immunity [10]. The increase in the use of herbal medicines in disease management has been attributed to many factors, including decreased side effects [11]. Recently, there has been a resurgence of interest in polyherbal formulations for treating different chronic illnesses [12]. During polyherbal formulation, different herbs are combined for efficacy reinforcement, potentiation, and antagonism and to suppress the toxicity of individual herbs without diminishing efficacy [12]. Since the pathogenesis of liver disease and immune suppression involve a cascade of events triggered by many complex factors, combining different herbs that possess different sites of action against such diseases could provide a potentially potent remedy [12].

Liver disease starts with inflammation caused by a virus, drug, alcohol, an autoimmune disease, or a diet similar to one containing monosodium glutamate [6,13]. If inflammation is repetitive, liver cells attempt to repair themselves and, in so doing, form scar tissues called fibrosis [13]. Fibrosis disrupts the main functions of the liver, such as blood detoxification. The accumulation of fibrosis can result in cirrhosis if it is poorly managed [14]. For liver fibrosis diagnosis, the gold standard is a liver biopsy [15]. Common laboratory findings of liver damage include elevated serum bilirubin and elevated liver enzymes such as aspartate aminotransferase (AST) and alkaline phosphatase (ALP). For treatment, liver fibrosis is generally reversible [15]. Treatment of underlying causes with antivirals, anti-inflammatory drugs, alcohol consumption cessation, behavioral modifications, and antifibrotic drugs such as pirfenidone can prevent progressive fibrosis [14]. At the cirrhosis stage, a liver transplant may be necessary.

Globally, for every 25 deaths that occur worldwide, one is liver related [16]. The current global prevalence of liver fibrosis ranges between 3.6 and 13.0% [17]. In Uganda, the prevalence of significant liver fibrosis among HIV-negative patients is greater than that among HIV-positive patients (24% vs. 14%) [18]. Liver fibrosis is also known for disrupting the immune system and, if left unattended to, can suppress the immune system [19].

A suppressed immune system increases susceptibility to infections. Some people with immune deficiencies can be undiagnosed for several years. Immune dysfunction can be divided into four categories: autoimmunity, allergy, immune suppression, and inflammatory dysfunction [20]. Inflammatory dysfunction contributes to liver diseases [13]. The magnitude of inflammation is regulated by both the innate and adaptive immune systems [20]. Severe anomalies in the immune system of rats can result from inflammatory responses triggered by splenic injury caused by monosodium glutamate [21]. The white blood cell (WBC) count and neutrophil-to-lymphocyte ratio (NLR) predict systemic inflammatory responses. A high neutrophil-to-lymphocyte ratio suggests dysregulation of immune and inflammatory responses [22]. Other indicators of inflammation include lymphocyte and neutrophil counts [22]. Neutrophils are part of innate immunity. Moreover, lymphocytes are components of the adaptive immune system. Chronic low-grade inflammation follows chronic illnesses; hence, lifestyle changes, in some chronic diseases, are needed to reduce inflammation and improve health [23]. Additionally, there is a need to develop safe, affordable, and effective anti-hepatic fibrosis and immune-boosting drugs, which can be conventional or herbal formulations.

The Immuno-Kachiks polyherbal formulation is among the many available traditional herbal preparations created to boost the immune system and reverse liver fibrosis. Pharmacist Kachiko Geoffrey, a well-known traditional Ugandan medicine practitioner, invented the Immuno-Kachiks formula. Herbs with reported immune stimulatory and anti-hepatic fibrosis potential were selected and combined during formulation. The composition of IKF included *Artemisia annua* [24], *Andrographis paniculata* [25], *Vernonia amygdalina* [26], *Centella asiatica* [27], *Phyllanthus reticulatus* [28,29], *Portulaca oleracea* [30], and *Annona muricata* [31]. Extracts obtained from each of those herbs were combined.

The history of Chinese medicine has led to the documentation of the practice of combining herbal drugs. Combining herbs with reported activity does not always guarantee efficacy. Some combinations are antagonistic, while others potentiate toxicity [12]. Therefore, this study aimed to determine HPLC fingerprints and efficacy of the Immuno-Kachiks polyherbal formulation in reversing monosodium glutamate-induced liver damage and immune suppression in male Wistar rats.

## 2 Materials and methods

### 2.1 Materials

The Wildcrafted plant material included leaves of *Artemisia annua*, shoots of *Andrographis paniculata*, leaves of *Vernonia amygdalina,* shoots of *Centella asiatica*, leaves of *Phyllanthus reticulatus*, shoots of *Portulaca oleracea*, and leaves of *Annona muricata*. The collected herbal materials were identified and authenticated (ref: ***Must/Pharm/007***) by a taxonomist, Dr. Olet Eunice Apio, at the Department of Botany, Mbarara University of Science and Technology, and further confirmed using www.worldfloraonline.org.

### 2.2 Preparation of polyherbal formulation

The IKF is composed of seven Ugandan medicinal herbs. The experiment followed standardized preparatory processes. All the plant materials were dried under shade except for the 1 kg of fresh shoots of *Portulaca oleracea, which were* juiced directly. Two hundred grams (200 g) of dried leaves of *Artemisia annua*, shoots of *Andrographis paniculata*, leaves of *Vernonia amygdalina,* shoots of *Centella asiatica*, leaves of *Phyllanthus reticulatus*, and leaves of *Annona muricata* were each separately immersed in 1 L of 50% ethanol and then macerated for 72 h. The filtrate was collected in clean glass beakers. Evaporation of the condensed extract of each herbal filtrate was performed to a concentration of approximately 2 to 1 g/mL. We placed each concentrate in a dehydrator. The obtained dry extracts of *Portulaca oleracea*, *Artemisia annua*, *Andrographis paniculata*, *Vernonia amygdalina, Centella asiatica*, *Phyllanthus reticulatus*, and *Annona muricata* were finely ground and measured before being combined at a ratio of 2.5**:** 3.5**:** 2**:** 2**:** 1.5**:** 1**:** 3. The study used an accepted formulation (the *Immuno-Kachiks formula*).

### 2.3 Chromatographic analysis of the Immuno-Kachiks formula

IKF at a concentration of 1000 μg/mL was prepared in water and vortexed. The solution was prepared following isocratic elution in a reversed-phase HPLC assay at a flow rate of 1.0 mL/min, a column temperature of 30 °C, an injection volume of 20 μL, and a mobile phase of deionized water/methanol at a ratio of 7:3 delivered by pumps A and B, respectively, and detected at wavelengths of 230, 254 and 370 nm. The acquisition time for each injection was 60 min. The LC-Solution Software was used to process the obtained data.

We performed high-performance liquid chromatography (HPLC) analysis on a UFLC Prominence Shimadzu chromatograph (Japan) at the Pharmaceutical Chemistry/Analytical Laboratory, Mbarara University of Science and Technology, Uganda. The HPLC instrument used consisted of a UV–visible detector (SPD-20A), an autosampler SIL-20AC HT, an LC-20 AD pump, a column oven (CTO-20AC), and an online degassing unit (DGU-20A).

### 2.4 Monosodium glutamate-induced hepatic damage and immune suppression study

#### 2.4.1 Experimental animals

The study used healthy male Wistar rats aged 10–12 weeks. The Wistar rats were fed commercial pellets and water ad libitum. The rats were allowed to acclimatize to the laboratory environment for eight days before the commencement of the study. During the entire study, all the animals were kept in wooden cages and maintained at a room temperature ranging from 21 to 28 °C and a relative humidity ranging from approximately 60 to 70%. All rats were allowed access to a natural dark-light cycle and a standard diet. Animals were handled following the ARRIVE guidelines. The study was approved by the institutional animal care committee of Mbarara University of Science and Technology (ref: **MUST-2021**–**312**) and registered at the Uganda National Council for Science and Technology (ref: **HS1926ES**).

#### 2.4.2 Drugs and reagents

Five hundred grams of monosodium glutamate (Ajinomoto) was purchased from Ajinomoto Co., Tokyo, Japan. We used only analytical-grade chemicals and solvents during the study.

#### 2.4.3 Induction of hepatic damage and immune suppression

Except for the rats in the normal control group, all the experimental animals were orally administered monosodium glutamate at a dose of 2000 mg/kg dissolved in distilled water daily for 12 weeks. The control group animals received 2 mL of distilled water orally. At the end of the induction period, six rats from each group were used to confirm successful induction. Blood and liver samples were collected. Histomorphological alterations in the liver parenchyma confirmed liver damage. Hematological indices were analyzed to verify immune suppression and liver damage.

#### 2.4.4 Study design

We calculated the sample size using the Resource Equation method [32]. The study included 15 male albino Wistar rats equally divided as follows: Group I: the normal control group without induction; Group II: the disease control group with induced rats; and Groups III, IV, and V: groups of disease-induced rats treated with Immuno-Kachiks formulation at doses of 400 mg/kg (low dose), 800 mg/kg (medium dose) and 1500 mg/kg (high dose) body weight, respectively.

The study design involved 12 weeks of monosodium glutamate administration to induce liver disease and immune suppression. After 12 weeks, curative treatment with IKF was administered for the next 28 days. We measured the body weights of the animals at the beginning of the study and weekly thereafter. On the last day of the experiment, we evaluated biochemical indices and studied changes in the liver histomorphology. Liver damage was evaluated by measuring the liver weight-to-body weight ratio, AFP concentration, total bilirubin concentration, alkaline phosphatase (ALP) level, and aspartate transaminase (AST) activity. Additionally, the collected livers were subjected to histopathological studies [15]. The WBC, NLR, lymphocyte, neutrophil, and mixed cell counts were used to evaluate the immune response [22]. These evaluations assessed the efficacy of the polyherbal formulation.

#### 2.4.5 Parameters evaluated

a) **Physical parameters:** We recorded all animal body and liver weights during the study.
b) **Biochemical indices of liver function:** The collected blood samples were allowed to clot before the serum was separated for 15 min at 2500 rpm. We evaluated the serum AST, total bilirubin, and ALP levels using spectrophotometric diagnostic kits [33].

Alpha-fetoprotein (AFP) was measured with an enzyme-linked immunosorbent assay (ELISA) kit, and all the measurements were performed in triplicate [34].

c) **Biochemical indices of immune function:** To determine the total and differential white blood counts in the collected blood samples, we used an automated hematology analyzer (HB-7021 Hematology Analyzer) [35].
d) **Histopathology of the liver:** On the last day of the study, the liver of each rat was removed, cleaned, and weighed. The livers were fixed in a 10% formalin solution and embedded in paraffin blocks. Carefully cut and stained tissue sections were subjected to histopathological evaluation. A histopathologist analyzed the different prepared slides under a light microscope and noted alterations in the liver parenchyma [33].
e) **Statistical analysis:** The quantitative data obtained were stored in Microsoft Excel and subsequently transferred to STATA version 12.1 software for analysis. The data were analyzed via two-way analysis of variance (ANOVA) and the one-sample *t*-test. The Bonferroni comparison test was used to analyze all the results via ANOVA. The data are presented as the mean ± standard deviation. A p-value ≤0.05 was considered to indicate statistical significance.

## 3 Results

### 3.1 Chemical profile of IKF by HPLC-UV

**Fig. 1:**
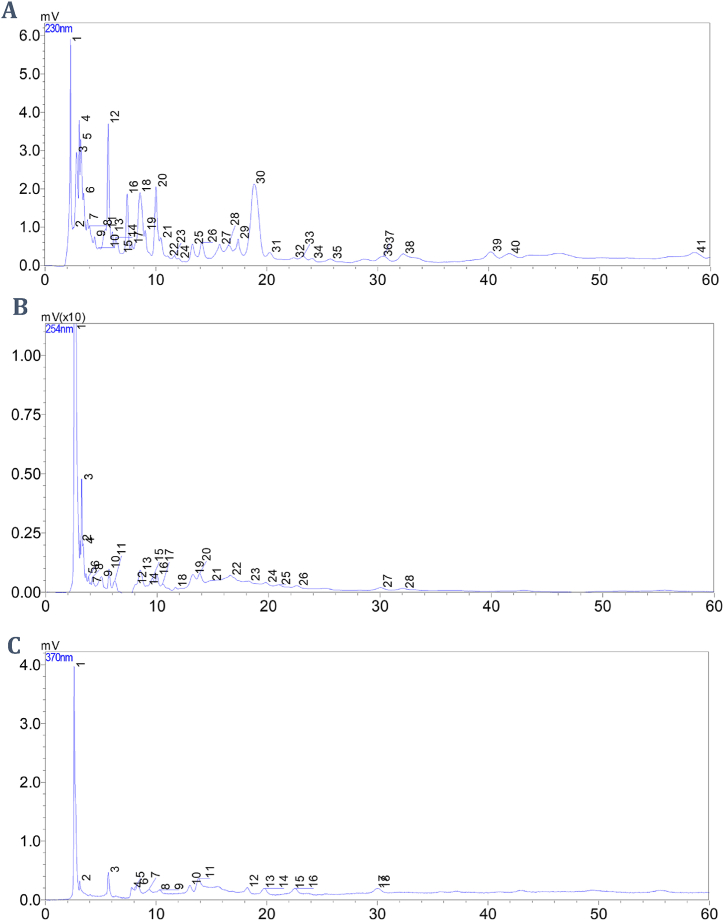
Base peak intensity chromatographs of IKF at 230 nm (A), 254 nm (B), and at 370 nm (C).

Forty-one (41), 28, and 18 components of IKF were detected at 230 nm, 254 nm, and 370 nm wavelengths, respectively (Fig. 1 and Appendix Table 3. A, Table 3. B, & Table 3. C.). At each wavelength (230 nm, 254 nm, and 370 nm), four unique peaks were selected for the identification of IKF. The materials had identical retention times (min), as shown in Fig. 1 and Table 1. The four similar peaks (peaks 1, 2, 3, and 4) at each wavelength are shown.

**Table 1:** Peaks and their retention times at 230 nm, 254 nm, and 370 nm.

Peak name (peak number at wavelength: 230 nm, 254 nm, & 370 nm, respectively)	Retention time (Min)
Peak 1 (2,1,1)	2.58 ± 0.02
Peak 2 (4,2,2)	3.08 ± 0.01
Peak 3 (12, 10, 3)	5.68 ± 0.01
Peak 4 (32,26,15)	22.44 ± 0.06

The data are expressed as the mean ± S.E.M. of similar retention times at three different wavelengths: 230 nm, 254 nm, and 370 nm. The peak numbers are similar to those in Fig. 1A, B, and C.

### 3.2 Monosodium glutamate-induced hepatic damage and immune suppression study

#### 3.2.1 Effect of IKF on the body weight-to-liver weight ratio

Table-2 shows that monosodium glutamate had no significant effect on the liver-to-body weight ratio in the Wistar rats. Similarly, treatment with IKF also had no significant impact on the liver-to-body weight ratio except in the TG1-400 group, where a significant increase in the liver-to-body weight ratio occurred.

**Table-2:** Effect of polyherbal formulation on liver to body weight ratio and biochemical indices in monosodium glutamate intoxicated rats.

Parameters	NC	DC	TG1-400	TG2-800	TG3-1500
Liver/Body ratio	0.032 ± 0.0022	0.035 ± 0.0021	0.039 ± 0.0024^a^^,^ **	0.037 ± 0.0027	0.036 ± 0.0023
AFP in ng/mL	26 ± 1.0	27 ± 0.45	24 ± 5.9	27 ± 0.15	28 ± 0.55
AST in U/L	141 ± 14	131 ± 11	176 ± 53	211 ± 16**	193 ± 39*
ALP in U/L	61 ± 1.6	89 ± 20^a^^,^ *	94 ± 26	120 ± 16^c^^,^ *	136 ± 27^d^^,^ *
Total Bilirubin	0.65 ± 0.05	0.58 ± 0.09	0.59 ± 0.26	0.92 ± 0.32	0.94 ± 0.07^d^^,^ **
WBC (109/L)	5.2 ± 1.6	7.3 ± 2.1a, ns	7.1 ± 1.0	9.3 ± 2.3	4.9 ± 0.6
Lymphocytes (%)	79 ± 6.6	57 ± 7.0^a^^,^ **	67 ± 14	75 ± 11^c^^,^ *	85 ± 5.6^d^^,^ **
Neutrophils (%)	17 ± 7.1	39 ± 8.0^a^^,^ *	30 ± 14	29 ± 11	7.8 ± 1.3^d^^,^ **
Mixed Cell Count (%)	4.4 ± 0.59	4.5 ± 1.1	3.7 ± 0.59	4.6 ± 0.76	3.7 ± 0.34

Values are expressed as the mean ± S.E.M for n = 3. *P < 0.05, **P < 0.01, ns-nonsignificant. NC: Normal control, DC: Disease control, TG1-400: Polyherbal formulation 400 mg/kg, TG2-800: Polyherbal formulation 800 mg/kg, TG3-1500: Polyherbal formulation 1500 mg/kg, AFP: Alpha fetoprotein, AST: Aspartate aminotransferase, ALP: Alkaline phosphatase, WBC: White blood cells.

^b^ DC vs TG1-400.

a NC vs DC.

c DC vs TG2-800.

d DC vs TG2-1500.

#### 3.2.2 Effect of IKF on serum biochemical indices of liver function

Despite the cessation of monosodium glutamate administration for 28 days, the serum ALP concentrations remained significantly high at the end of the study period. Treatment with 800 mg/kg or 1500 mg/kg IKF significantly increased the AST and ALP serum levels. Only the 1500 mg/kg dose of IKF significantly increased total serum bilirubin, as shown in Table-2. At the end of the experiment, there was no significant difference in the serum alpha-fetoprotein levels between the control and treatment groups, as shown in Table-2.

#### 3.2.3 Effect of IKF on serum biochemical indices of immune function

White blood cell and mixed cell counts were not significantly different between the control group and treatment group, as shown in Table-2. Despite the cessation of monosodium glutamate administration for the last 28 days of the experiment, the neutrophil level significantly increased, while the lymphocyte level markedly decreased by the end of the study period. Conversely, treatment with IKF significantly increased the lymphocyte count and decreased the neutrophil count in the blood.

#### 3.2.4 Histopathological findings in liver tissues

Fig. 3 (A) shows normal macroscopic liver morphology across all the experimental groups. As shown in Fig. 3 B (DC), monosodium glutamate caused mild inflammation, early-stage fibrosis, hepatic steatosis, and hepatocyte swelling in the disease control (DC) group rats. Discontinuation of monosodium glutamate administration did not reverse hepatic steatosis or hepatocyte swelling after 28 days. Treatment with IKF restored the normal liver parenchyma of the rats (Fig. 3 B (TG1-3)). Additionally, Fig. 3 B (TG3-1500) cells exhibited mild drug-induced inflammation characterized by clusters of macrophages in the portal triad regions.

## 4 Discussion

Herbal formulation fingerprinting involves the application of high-performance liquid chromatography [36] Chromatographic fingerprinting is vital for standardizing and authenticating herbs and herbal formulations. In HPLC-UV/VIS, fingerprinting focuses on the obtained chromatogram and the retention time [36]. Even though the retention time is usually considered unique for each chemical within a sample, it is essential to note that peaks exhibiting identical retention times do not always indicate that the peaks are of similar compounds when using HPLC-fixed UV/Vis [36]. Retention time values are less preferred than relative retention time values, as relative retention time values minimize the effects of confounders during standardization [36]. Since the relative retention time formula requires a standard retention time, this study generated standard retention times. Future samples exhibiting similar relative retention times (Table 1) and HPLC chromatograms (Fig. 1) will be genuine IKF products.

Animal studies have demonstrated that monosodium glutamate induces liver necrosis and liver fibrosis. Additionally, monosodium glutamate increases aspartate aminotransferase, alkaline phosphatase, and total bilirubin levels in the blood circulation [37]. Our study findings revealed that halting the ingestion of monosodium glutamate for 28 days does not restore the normal level of alkaline phosphatase. Based on the normal alkaline phosphatase concentration ranging from 56.8 to 128 U/L [38], the increase in alkaline phosphatase activity caused by IKF was mild. High alkaline phosphatase levels may indicate liver damage [39]. The bones and the biliary system also contain the alkaline phosphatase enzyme [39]. Hence, increased alkaline phosphatase levels not only indicate liver damage but also bone disease. Because of the presence of alkaline phosphatase in the biliary system, cholestasis (slow or blockage of bile flow) increases pressure in the biliary system, causing damage to the cells lining the bile ducts and resulting in leakage of alkaline phosphatase enzymes into the blood [39]. Since hepatocytes lack alkaline phosphatase enzymes, hepatocyte damage does not increase ALP levels in the blood.

In contrast to most similar studies, doses of IKF equal to or greater than 800 mg/kg significantly increase the serum levels of aspartate aminotransferase, alkaline phosphatase, and total bilirubin. Several other studies have also reported drug-induced increases in the serum levels of total bilirubin, alkaline phosphatase, and aspartate aminotransferase [40]. An aspartate aminotransferase (AST) enzyme test is not specific for liver damage. Muscle damage can also cause an increase in aspartate aminotransferase levels in the blood. As mentioned earlier, doses of IKF equal to or greater than 800 mg/kg also significantly increased the serum levels of total bilirubin. A high serum bilirubin concentration is regarded as an indicator of actual liver function because it signifies the inability of the liver to reuptake and process bilirubin into bile [41].

Interestingly, studies have revealed that both mildly and moderately elevated serum bilirubin are beneficial since bilirubin is a potent antioxidant [41]. Antioxidants are known to reverse liver damage. Liver damage can also be detected using other biomarkers, such as the level of serum alpha-fetoprotein. Earlier studies considered alpha-fetoprotein to be highly specific for hepatocarcinoma. A study by Turshudzhyan and Wu in 2022 revealed that the alpha-fetoprotein test has a sensitivity of 60% and a specificity of 30% [42]. Elevated alpha-fetoprotein levels do not always indicate liver cancer. Increased alpha-fetoprotein levels can also indicate other cancers, including testicular, stomach, and bowel cancer. In the present study, neither monosodium glutamate nor IKF significantly raised the alpha-fetoprotein levels above or below our experimental normal. The absence of a considerable effect indicated that neither monosodium glutamate nor IKF might be carcinogenic. Our findings further support the findings of other studies that have concluded that monosodium glutamate is not carcinogenic.

Hematological indices are used to assess the potential benefit and toxicity of herbal formulations to the body [15,22]. Hematological parameters commonly evaluated to establish one's immune system's status include white blood cell (WBC) count, lymphocyte count, and neutrophil count [21]. Interestingly, IKF did not seem to have a significant effect on the white blood cell count but significantly altered the number of differential white blood cell counts in the blood (Table-2), suggesting that it has immunostimulatory potential in rats. Some studies have indicated that the nutrient elements in herbs are responsible for their potential to increase white blood cells in the blood [43].

Notably, the blood of both rats and mice usually contains more lymphocytes than neutrophils, while the blood of humans is typically richer in neutrophils than lymphocytes [44]. This key variation lays the foundation for our discussion below. Notably, in the present study, monosodium glutamate significantly decreased the number of lymphocytes and increased the neutrophil count. This observation suggested that monosodium glutamate increases innate immunity while suppressing adaptive immunity. This pattern exhibited by monosodium glutamate has negative consequences. A low lymphocyte count in the blood indicates systemic inflammation and is associated with liver disease [45]. A high neutrophil count induces liver fibrogenesis [46]. Neutrophilia indicates acute inflammation. Other researchers have reported adaptive immune suppression by monosodium glutamate. In 2022, Das et al. reported that monosodium glutamate was a silent immune killer that increased oxidative stress within lymphocytes [21]. Additionally, Jovic et al. 2009 demonstrated that glutamate causes lymphocyte apoptosis. The above findings could explain the low lymphocyte count we observed and could subsequently further explain our histopathological findings. IKF significantly increased the level of lymphocytes in the blood while simultaneously reducing the level of neutrophils back to the normal range. This observation suggested that IKF can potentially boost the immune system of Wistar rats. A differential white blood cell count characterized by a high neutrophil count is associated with all sorts of mortality [47]. A high neutrophil-to-lymphocyte ratio exacerbates liver-related mortality [47]. The neutrophil-to-lymphocyte ratio has proven to be a simple and effective parameter for assessing systemic inflammatory status.

**Fig. 2:**
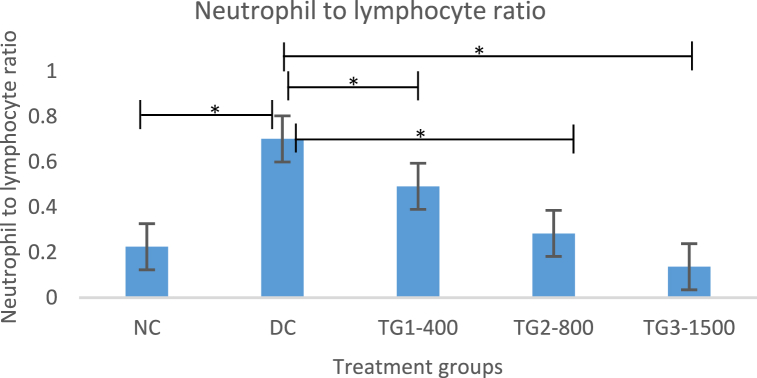
Analysis of the neutrophil-to-lymphocyte ratio of Wistar rats with immunity suppressed by monosodium glutamate. The expressed values are presented as the means ± S.E.M.s for n = 3. *P < 0.05. NC: normal control, DC: disease control, TG1-400: polyherbal formulation 400 mg/kg, TG2-800: polyherbal formulation 800 mg/kg, TG3-1500: polyherbal formulation 1500 mg/kg.

Inflammation causes fibrosis of the liver. Liver fibrosis stage is directly and significantly associated with the neutrophil-to-lymphocyte ratio. The neutrophil-to-lymphocyte ratio is a prognostic indicator for assessing chronic liver disease [48]. During our study, monosodium glutamate significantly increased the neutrophil-to-lymphocyte ratio (Fig. 2), which suggested that monosodium glutamate potentially caused inflammation in the body of rats. Our observations agreed with those of other studies reporting glutamate as an excitotoxin [5,9]. Excitotoxins can also damage nerve cells, causing inflammation [5,9]. Based on our study findings, halting the administration of monosodium glutamate for 28 days did not significantly increase the neutrophil-to-lymphocyte ratio (Fig. 2). In 2022, Joshi et al. reported a significant correlation between a high neutrophil-to-lymphocyte ratio and disease severity [49]. Treatment with IKF significantly reduced the neutrophil-to-lymphocyte ratio. These findings suggested that IKF could be a potential remedy for chronic inflammation.

**Fig. 3:**
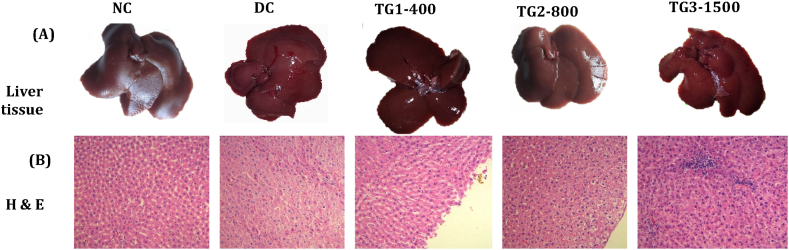
Effect of IKF on monosodium glutamate-induced hepatic steatosis and hepatocyte swelling in Wistar rats. (A) Macroscopic pictures of the livers; (B) Microscopic photos of hematoxylin & eosin (H&E) stained liver tissues. NC: Normal control, DC: Disease control, TG1-400: Polyherbal formulation 400 mg/kg, TG2-800: Polyherbal formulation 800 mg/kg, TG3-1500: Polyherbal formulation 1500 mg/kg.

The livers of the diseased control (DC) group that received only monosodium glutamate had histopathological lesions similar to those reported in other studies. IKF improved the liver histoarchitecture of the monosodium glutamate-challenged rats (Fig. 3). Like other drugs, IKF also induced mild inflammation at 1500 mg/kg.

## 5 Conclusion

Our findings highlight the significant role of monosodium glutamate consumption in immune suppression and liver damage. Additionally, IKF potentially boosts suppressed adaptive immunity and can reverse early-stage liver fibrosis in rats. This research scientifically confirmed the curative potential, immunostimulatory potential, and HPLC fingerprints of the Immuno-Kachiks formula for early-stage liver fibrosis for the first time.

## Funding

10.13039/100004421The World Bank and the Government of Uganda through Pharm-Biotechnology and Traditional Medicine (PHARMBIOTRAC) (**P151847**), an Africa Higher Education Center of Excellence (ACEII) at the 10.13039/501100009915Mbarara University of Science and Technology (10.13039/501100009915MUST), and Uganda and PHARMBIOTRAC Incubation Hub supported this work. The funder was involved neither in the study design nor in the study implementation.

## Declaimer

This article presents the findings of the postgraduate research thesis submitted to Mbarara University of Science and Technology, Mbarara, Uganda.

## CRediT authorship contribution statement

**Geoffrey Kachiko:** Writing – original draft, Methodology, Investigation, Conceptualization. **Anke Weisheit:** Writing – review & editing. **Clement Olusoji Ajayi:** Validation, Resources, Investigation. **Casim Umba Tolo:** Writing – review & editing. **Jonans Tusiimire:** Supervision.

## Declaration of competing interest

The authors declare that they have no known competing financial interests or personal relationships that could have appeared to influence the work reported in this paper.
