# Supplementary material for: Immuno-Kachiks formula immunomodulates and ameliorates hepatic damage induced by monosodium glutamate in rats
Source: Heliyon. 2024 Feb 29;10(5):e27076. doi: 10.1016/j.heliyon.2024.e27076 (PMC10920360; doi:10.1016/j.heliyon.2024.e27076)
Supplement: Multimedia component 1 [file mmc1.docx]

**Table 3.A:** Chemical profile of *Immuno-Kachik* herbal formulation by HPLC at 230nm

| Peak# | Ret. Time | Area | Height | Peak Start | Peak End | Area% | USP Width | Width (50%) | Resolution |
| --- | --- | --- | --- | --- | --- | --- | --- | --- | --- |
| 1 | 2.290 | 57202 | 5930 | 1.642 | 2.492 | 7.5500 | 0.161 | 0.078 | -- |
| 2 | 2.550 | 5996 | 1020 | 2.492 | 2.592 | 0.7913 | 2.125 | 0.000 | 0.227 |
| 3 | 2.837 | 50432 | 2973 | 2.592 | 3.008 | 6.6564 | 0.536 | 0.000 | 0.216 |
| 4 | 3.077 | 25260 | 3808 | 3.008 | 3.158 | 3.3340 | 0.258 | 0.000 | 0.604 |
| 5 | 3.215 | 41472 | 3309 | 3.158 | 3.425 | 5.4738 | 0.491 | 0.000 | 0.367 |
| 6 | 3.478 | 24173 | 1900 | 3.425 | 3.717 | 3.1905 | 0.785 | 0.000 | 0.412 |
| 7 | 3.826 | 14289 | 1197 | 3.717 | 3.942 | 1.8860 | 0.768 | 0.000 | 0.448 |
| 8 | 4.013 | 19063 | 1033 | 3.942 | 4.333 | 2.5161 | 1.551 | 0.000 | 0.161 |
| 9 | 4.466 | 13775 | 746 | 4.333 | 4.733 | 1.8181 | 0.763 | 0.000 | 0.392 |
| 10 | 4.832 | 7641 | 455 | 4.733 | 5.025 | 1.0085 | 2.842 | 0.000 | 0.203 |
| 11 | 5.445 | 21208 | 939 | 5.025 | 5.517 | 2.7992 | 1.715 | 0.000 | 0.269 |
| 12 | 5.692 | 50270 | 3685 | 5.517 | 5.975 | 6.6350 | 0.333 | 0.186 | 0.241 |
| 13 | 6.067 | 10387 | 855 | 5.975 | 6.233 | 1.3710 | 0.559 | 0.000 | 0.841 |
| 14 | 6.385 | 16995 | 707 | 6.233 | 6.808 | 2.2432 | 0.833 | 0.000 | 0.457 |
| 15 | 6.908 | 2526 | 281 | 6.808 | 6.958 | 0.3334 | 8.945 | 0.000 | 0.107 |
| 16 | 7.408 | 33595 | 1832 | 6.958 | 7.683 | 4.4342 | 0.413 | 0.234 | 0.107 |
| 17 | 7.834 | 8047 | 515 | 7.683 | 7.983 | 1.0621 | 0.843 | 0.000 | 0.679 |
| 18 | 8.559 | 61103 | 1853 | 7.983 | 8.933 | 8.0649 | 0.823 | 0.493 | 0.869 |
| 19 | 9.033 | 17447 | 858 | 8.933 | 9.608 | 2.3028 | 0.678 | 0.000 | 0.632 |
| 20 | 10.000 | 39170 | 1988 | 9.608 | 10.308 | 5.1699 | 0.471 | 0.270 | 1.685 |
| 21 | 10.441 | 14114 | 650 | 10.308 | 10.917 | 1.8629 | 1.082 | 0.000 | 0.568 |
| 22 | 11.046 | 3514 | 166 | 10.917 | 11.333 | 0.4638 | 3.429 | 0.000 | 0.268 |
| 23 | 11.666 | 4120 | 163 | 11.333 | 11.900 | 0.5438 | 11.998 | 0.000 | 0.080 |
| 24 | 12.021 | 1695 | 95 | 11.900 | 12.383 | 0.2237 | 0.935 | 0.000 | 0.055 |
| 25 | 13.280 | 10358 | 433 | 12.767 | 13.758 | 1.3671 | 0.585 | 0.336 | 1.657 |
| 26 | 14.128 | 10471 | 440 | 13.758 | 14.625 | 1.3820 | 0.635 | 0.365 | 1.391 |
| 27 | 15.738 | 12114 | 372 | 14.950 | 16.092 | 1.5989 | 1.026 | 0.597 | 1.940 |
| 28 | 16.525 | 13273 | 345 | 16.092 | 16.933 | 1.7519 | 1.710 | 0.000 | 0.575 |
| 29 | 17.409 | 14918 | 517 | 16.933 | 17.892 | 1.9690 | 0.592 | 0.376 | 0.768 |
| 30 | 18.856 | 101456 | 1959 | 17.892 | 19.883 | 13.3910 | 1.291 | 0.796 | 1.537 |
| 31 | 20.256 | 5405 | 172 | 19.883 | 20.917 | 0.7134 | 0.933 | 0.516 | 1.259 |
| 32 | 22.402 | 1225 | 50 | 22.025 | 22.742 | 0.1617 | 0.682 | 0.417 | 2.659 |
| 33 | 23.250 | 2817 | 92 | 22.742 | 23.742 | 0.3718 | 0.841 | 0.496 | 1.114 |
| 34 | 24.069 | 1410 | 59 | 23.742 | 24.550 | 0.1862 | 0.654 | 0.395 | 1.097 |
| 35 | 25.760 | 2842 | 71 | 25.108 | 26.325 | 0.3752 | 1.045 | 0.638 | 1.990 |
| 36 | 30.342 | 2651 | 114 | 29.658 | 30.392 | 0.3499 | 2.821 | 0.000 | 2.370 |
| 37 | 30.499 | 3621 | 121 | 30.392 | 31.225 | 0.4779 | 1.784 | 0.000 | 0.069 |
| 38 | 32.294 | 6221 | 141 | 31.525 | 33.025 | 0.8211 | 1.156 | 0.682 | 1.221 |
| 39 | 40.181 | 11073 | 204 | 39.208 | 40.908 | 1.4615 | 1.417 | 0.824 | 6.131 |
| 40 | 41.847 | 7321 | 120 | 40.908 | 42.692 | 0.9663 | 1.546 | 0.971 | 1.125 |
| 41 | 58.572 | 6974 | 121 | 57.833 | 59.517 | 0.9205 | 1.621 | 0.956 | 10.563 |

**Table 3.B:** Chemical profile of Immuno-Kachik herbal formulation by HPLC at 254 nm wavelength

| Peak# | Ret. Time | Area | Height | Peak Start | Peak End | Area% | USP Width | Width (50%) | Resolution |
| --- | --- | --- | --- | --- | --- | --- | --- | --- | --- |
| 1 | 2.589 | 410940 | 43363 | 2.000 | 3.150 | 59.1238 | 0.181 | 0.093 | -- |
| 2 | 3.087 | 2312 | 685 | 3.033 | 3.150 | 0.3327 | 0.101 | 0.055 | 3.529 |
| 3 | 3.221 | 31581 | 4841 | 3.150 | 3.317 | 4.5437 | 0.212 | 0.108 | 0.854 |
| 4 | 3.349 | 18912 | 2100 | 3.317 | 3.542 | 2.7210 | 0.796 | 0.000 | 0.255 |
| 5 | 3.610 | 7896 | 838 | 3.542 | 3.733 | 1.1360 | 0.528 | 0.000 | 0.393 |
| 6 | 3.831 | 7470 | 715 | 3.733 | 3.950 | 1.0747 | 0.467 | 0.000 | 0.444 |
| 7 | 4.004 | 3434 | 465 | 3.950 | 4.083 | 0.4941 | 0.831 | 0.000 | 0.268 |
| 8 | 4.183 | 10570 | 696 | 4.083 | 4.458 | 1.5207 | 0.691 | 0.000 | 0.235 |
| 9 | 4.995 | 28019 | 738 | 4.458 | 5.492 | 4.0313 | 0.949 | 0.554 | 0.990 |
| 10 | 5.674 | 15462 | 1076 | 5.492 | 5.942 | 2.2246 | 0.363 | 0.204 | 1.034 |
| 11 | 6.189 | 16903 | 566 | 5.942 | 7.325 | 2.4319 | 0.698 | 0.449 | 0.971 |
| 12 | 8.040 | 6820 | 418 | 7.383 | 8.092 | 0.9813 | 1.863 | 0.000 | 1.445 |
| 13 | 8.493 | 31942 | 994 | 8.092 | 8.950 | 4.5957 | 0.756 | 0.512 | 0.346 |
| 14 | 9.090 | 4257 | 312 | 8.950 | 9.183 | 0.6125 | 3.381 | 0.000 | 0.288 |
| 15 | 9.574 | 17210 | 595 | 9.183 | 9.800 | 2.4761 | 1.095 | 0.000 | 0.216 |
| 16 | 9.991 | 13806 | 701 | 9.800 | 10.283 | 1.9863 | 0.612 | 0.000 | 0.488 |
| 17 | 10.491 | 9821 | 307 | 10.283 | 11.325 | 1.4130 | 1.023 | 0.000 | 0.612 |
| 18 | 11.654 | 1471 | 104 | 11.367 | 11.867 | 0.2116 | 0.394 | 0.224 | 1.643 |
| 19 | 13.200 | 16233 | 502 | 12.408 | 13.542 | 2.3355 | 0.981 | 0.000 | 2.250 |
| 20 | 13.810 | 12241 | 472 | 13.542 | 14.308 | 1.7612 | 0.786 | 0.000 | 0.691 |
| 21 | 14.733 | 1408 | 62 | 14.308 | 14.792 | 0.2026 | 1.087 | 0.000 | 0.987 |
| 22 | 16.542 | 11760 | 236 | 15.642 | 17.692 | 1.6920 | 1.470 | 0.802 | 1.415 |
| 23 | 18.215 | 1242 | 65 | 18.067 | 18.683 | 0.1786 | 0.702 | 0.000 | 1.540 |
| 24 | 19.738 | 2603 | 98 | 19.317 | 20.242 | 0.3745 | 0.700 | 0.426 | 2.173 |
| 25 | 20.928 | 1293 | 68 | 20.833 | 21.425 | 0.1860 | 0.782 | 0.000 | 1.606 |
| 26 | 22.513 | 2634 | 88 | 22.083 | 23.150 | 0.3790 | 0.824 | 0.493 | 1.974 |
| 27 | 30.059 | 4595 | 108 | 29.233 | 30.633 | 0.6611 | 1.143 | 0.686 | 7.673 |
| 28 | 32.042 | 2215 | 64 | 31.567 | 32.658 | 0.3186 | 0.975 | 0.564 | 1.873 |

**Table 3.C:** Chemical profile of Immuno-Kachik herbal formulation by HPLC at 370 nm

| Peak# | Ret. Time | Area | Height | Peak Start | Peak End | Area% | USP Width | Width  (50%) | Resolution |
| --- | --- | --- | --- | --- | --- | --- | --- | --- | --- |
| 1 | 2.591 | 52372 | 3972 | 2.133 | 5.517 | 55.0556 | 0.198 | 0.107 | -- |
| 2 | 3.088 | 1131 | 169 | 3.033 | 3.442 | 1.1885 | 0.163 | 0.087 | 2.753 |
| 3 | 5.678 | 5864 | 446 | 5.517 | 6.100 | 6.1643 | 0.318 | 0.177 | 10.776 |
| 4 | 7.774 | 2634 | 174 | 7.525 | 7.950 | 2.7691 | 0.918 | 0.000 | 3.393 |
| 5 | 7.987 | 1116 | 138 | 7.950 | 8.092 | 1.1734 | 1.652 | 0.000 | 0.165 |
| 6 | 8.321 | 5475 | 216 | 8.092 | 8.700 | 5.7551 | 1.111 | 0.000 | 0.242 |
| 7 | 9.356 | 3935 | 118 | 8.958 | 9.717 | 4.1369 | 1.882 | 0.000 | 0.692 |
| 8 | 10.267 | 1237 | 87 | 10.017 | 10.300 | 1.3003 | 0.888 | 0.000 | 0.657 |
| 9 | 10.329 | 1346 | 93 | 10.300 | 10.733 | 1.4150 | 0.613 | 0.000 | 0.083 |
| 10 | 13.065 | 2479 | 115 | 12.642 | 13.400 | 2.6063 | 0.556 | 0.330 | 4.684 |
| 11 | 13.849 | 5340 | 199 | 13.433 | 14.267 | 5.6137 | 0.713 | 0.428 | 1.236 |
| 12 | 18.245 | 2382 | 101 | 17.950 | 18.650 | 2.5038 | 0.645 | 0.407 | 6.475 |
| 13 | 19.722 | 1702 | 107 | 19.300 | 19.775 | 1.7893 | 1.626 | 0.000 | 1.301 |
| 14 | 19.808 | 1673 | 107 | 19.775 | 20.367 | 1.7589 | 0.962 | 0.000 | 0.067 |
| 15 | 22.400 | 1544 | 108 | 22.042 | 22.425 | 1.6228 | 0.993 | 0.000 | 2.652 |
| 16 | 22.567 | 2179 | 117 | 22.550 | 23.058 | 2.2902 | 1.507 | 0.000 | 0.133 |
| 17 | 29.892 | 1144 | 86 | 29.617 | 29.917 | 1.2025 | 0.647 | 0.000 | 6.800 |
| 18 | 30.136 | 1574 | 84 | 30.083 | 30.608 | 1.6543 | 1.421 | 0.000 | 0.237 |
